# The Association between Individual Food Groups, Limbic System White Matter Tracts, and Episodic Memory: Initial Data from the Aiginition Longitudinal Biomarker Investigation of Neurodegeneration (ALBION) Study

**DOI:** 10.3390/nu16162766

**Published:** 2024-08-19

**Authors:** Foteini Christidi, Archontoula Drouka, Dora Brikou, Eirini Mamalaki, Eva Ntanasi, Efstratios Karavasilis, Georgios Velonakis, Georgia Angelopoulou, Angeliki Tsapanou, Yian Gu, Mary Yannakoulia, Nikolaos Scarmeas

**Affiliations:** 1 First Department of Neurology, Aiginition Hospital, School of Medicine, National and Kapodistrian University of Athens, 11528 Athens, Greecegeorginangel@gmail.com (G.A.);; 2 Computational Neuroimaging Group (CNG), School of Medicine, Trinity College Dublin, D08 NHY1 Dublin, Ireland; 3 Department of Nutrition and Dietetics, Harokopio University, 17671 Athens, Greece; 4 Research Unit of Radiology and Medical Imaging, 2nd Department of Radiology, Attikon General University Hospital, School of Medicine, National and Kapodistrian University of Athens, 11528 Athens, Greece; 5 School of Medicine, Democritus University of Alexandroupolis, 68100 Alexandroupolis, Greece; 6 Taub Institute for Research in Alzheimer’s Disease and the Aging Brain, The Gertrude H. Sergievsky Center, Columbia University, New York, NY 10032, USA; yg2121@cumc.columbia.edu

**Keywords:** food groups, white matter tractography, limbic system, cognition, memory function, full-fat dairy, red meat, cold cuts, low-to-moderate alcohol, biomarkers

## Abstract

(1) Background: Many studies link food intake with clinical cognitive outcomes, but evidence for brain biomarkers, such as memory-related limbic white matter (WM) tracts, is limited. We examined the association between food groups, limbic WM tracts integrity, and memory performance in community-dwelling individuals. (2) Methods: We included 117 non-demented individuals (ALBION study). Verbal and visual episodic memory tests were administered, and a composite z-score was calculated. Diffusion tensor imaging tractography was applied for limbic WM tracts (fornix-FX, cingulum bundle-CB, uncinate fasciculus-UF, hippocampal perforant pathway zone-hPPZ). Food intake was evaluated through four 24-h recalls. We applied linear regression models adjusted for demographics and energy intake. (3) Results: We found significant associations between (a) higher low-to-moderate alcohol intake and higher FX fractional anisotropy (FA), (b) higher full-fat dairy intake and lower hPPZ FA, and (c) higher red meat and cold cuts intake and lower hPPZ FA. None of the food groups was associated with memory performance. (4) Conclusions: Despite non-significant associations between food groups and memory, possibly due to participants’ cognitive profile and/or compensatory mechanisms, the study documented a possible beneficial role of low-to-moderate alcohol and a harmful role of full-fat dairy and red meat and cold cuts on limbic WM tracts.

## 1. Introduction

Memory function is one of the cognitive functions that typically decline with age due to changes in brain structure and function, including reduced neurogenesis, synaptic plasticity, and neurotransmitter levels [1]. These memory changes can lead to mild cognitive impairment (MCI) [2], presented as amnestic MCI or multi-domain MCI. Dementia, particularly Alzheimer’s disease (AD), represents a more severe deterioration of memory function, marked by the accumulation of amyloid plaques and neurofibrillary tangles, which disrupt neural communication and lead to cell death [3]. This results in significant impairments in short-term memory, learning new information, and recalling previously known facts, severely impacting individuals’ daily functioning and quality of life [4].

The anatomical substrate of memory function involves various brain structures, such as the hippocampus [5] and white matter (WM) tracts of the limbic system, which are crucial for efficient information processing, storage, and retrieval [6,7]. WM tracts, such as the cingulum bundle (CB), the uncinate fasciculus (UF), the fornix (FX), and the hippocampal perforant pathway zone (hPPZ), play a significant role in memory function [8,9]. The CB connects the cingulate gyrus to the hippocampus, supporting episodic memory and spatial navigation [10,11]. The UF links the anterior temporal lobes to the frontal lobe, which is involved in semantic memory and emotional processing [10,11]. The FX is a major output tract of the hippocampus, which is essential for memory consolidation and recall [10,11]. The hPPZ, which connects the entorhinal cortex to the dentate gyrus of the hippocampus, is vital for the initial encoding and retrieval of episodic memories [12]. Damage or degeneration of these WM tracts, which is often observed in aging and neurodegenerative diseases like AD, can significantly impair memory function.

Both modifiable and non-modifiable factors are related to cognitive functions and brain health in aging and dementia [13], including memory function [14] and memory-related brain structures and WM tracts [15,16]. Diet is one of these modifiable risk factors, and different food groups are related to cognitive functions in healthy adults [17,18]. Regarding memory function, the consumption of fruits and vegetables has been associated with better verbal memory [19,20], whereas fish intake appears to have a beneficial role in memory as well [21]. Mixed results exist for dairy intake and memory functions, with some studies supporting a harmful role of milk intake on verbal memory performance [22] and others indicating a possible protective role of dairy in visuospatial memory [23] or incidence of AD [24]. Mixed evidence also exists on the association between red meat and episodic memory AD [25,26,27,28,29], with the largest prospective population study highlighting a negative association between processed red meat and memory performance [30]. Reports on the association between alcohol intake and memory function are still inconclusive, even though in a large prospective study moderate drinkers had higher verbal memory performance compared with non-drinkers [31].

While there have been many studies relating diet with clinical cognitive outcomes, investigations of the associations of diet with brain biomarkers are relatively fewer. Investigation of biomarkers may give us a glimpse of the biological pathways and a deeper understanding of the effects of diet on different cognitive systems. In recent years, some studies have started investigating the associations between diet and brain imaging biomarkers [32,33]. The association between the consumption of different food groups and gray matter (GM) volume, cortical thickness, or white matter hyperintensities (WMH) has been examined in previous studies: non-refined cereals [34,35], vegetables [34,35,36], fruits [34,35,37], fish [34,35,37,38], red meat [37,39], and alcohol [40,41,42,43,44,45]. It appears that non-refined cereals, vegetables, and fish have a positive or neutral effect on brain metrics, and red meat has a neutral or negative effect, whereas mixed results exist for fruits and alcohol intake. In contrast to GM volume and WMH, there is paucity of evidence regarding the association between food groups and WM integrity measured through diffusion tensor imaging (DTI), except for few studies on alcohol [43,46,47,48]. In addition, there is a lack of information on the associations between food groups and WM integrity in brain networks relating to memory function, possibly the most important domain of cognitive aging.

The present study aims to examine the role of different food groups on the integrity of limbic system WM tracts and on episodic memory in a group of community-dwelling non-demented adults followed in a cognitive clinic of a tertiary hospital as part of a longitudinal population study.

## 2. Materials and Methods

### 2.1. Participants and Study Design

Study participants were recruited from the Aiginition Longitudinal Biomarker Investigation of Neurodegeneration (ALBION) study [49], an ongoing longitudinal study. All participants provided informed consent for their participation in the study, which had been approved by the Institutional Review Board (IRB) of Aiginiton Hospital (225/10 May 2021). Non-demented individuals aged ≥ 40 years were included in the study. Although the majority of participants were cognitively intact, individuals with MCI were allowed in the study. Cognitive status was assessed following a detailed clinical assessment and a comprehensive neuropsychological and neuroimaging examination, as previously described in detail [50]. The present study includes ALBION participants in their baseline (first) evaluation. A total of 117 individuals of both sexes were included.

### 2.2. Neuropsychological Evaluation of Memory Function

Memory function was assessed using standardized cognitive tests, as part of a comprehensive neuropsychological evaluation that included screening tests of global cognition [Mini-Mental State Examination (MMSE) and Addenbrooke’s Cognitive Examination-revised (ACE-R)] and a variety of tests for attention, executive functions, memory function, language, and visuospatial dexterities. Memory function was examined based on the immediate and delayed recall of the Greek Verbal Learning Test (GVLT) and story recall (verbal episodic memory), as well as the immediate and delayed recall of the Medical College of Georgia Complex Figure Test (MCGCFT) (visual episodic memory). Raw scores on each test were transformed to z-scores using the mean and the standard deviation values derived from the healthy controls of the study group. A composite memory score was included in further analysis, calculated as the mean of the z-scores of verbal and visual episodic memory tests based on similar methodological approaches of previous studies [21]. All tests were administered by a trained neuropsychologist.

### 2.3. Neuroimaging Acquisition and Analysis

All participants underwent whole-brain imaging on a 3T Philips Achieva-Tx MR scanner (Philips, Best, The Netherlands) equipped with an eight-channel head coil. The imaging protocol included basic clinical- and research-used sequences. An experienced radiologist (G.V.) reviewed the sequences of the basic protocol [sagittal high-resolution 3D T1 weighted (3D-T1w), sagittal T2-fluid attenuation inversion recovery (T2-FLAIR), axial T2-TSE combined with fat suppression] to exclude unknown brain pathologies or severe cerebrovascular disease. For the purpose of the study, the following sequences were used: (a) a sagittal high-resolution 3D-T1w (acquisition voxel size 1.11 × 1.11 × 1.2 mm; TR = 6.7 ms; TE = 3.1 ms; TI = 2500 ms; flip angle = 9°), (b) an axial single-shot spin-echo echo-plannar imaging (EPI) DTI acquisition with 48 diffusion-encoding directions to evaluate the white matter integrity (acquisition voxel size 2 × 2 × 2 mm; 60 slices; two b factors with 0 s/mm^2^ (low b) and 1000 s/mm^2^ (high b); sensitivity-encoding reduction factor of 2; TR = 7576 ms; TE = 91 ms), and (c) a B0 reversed polarity gradient compared to the previous DTI acquisition using the same acquisition parameters to correct the geometrically distorted images due to EPI, during the post-processing process. An experienced MR physicist (E.K.) initially reviewed all sequences for major motion- or scanner-related artifacts.

**Figure 1:**
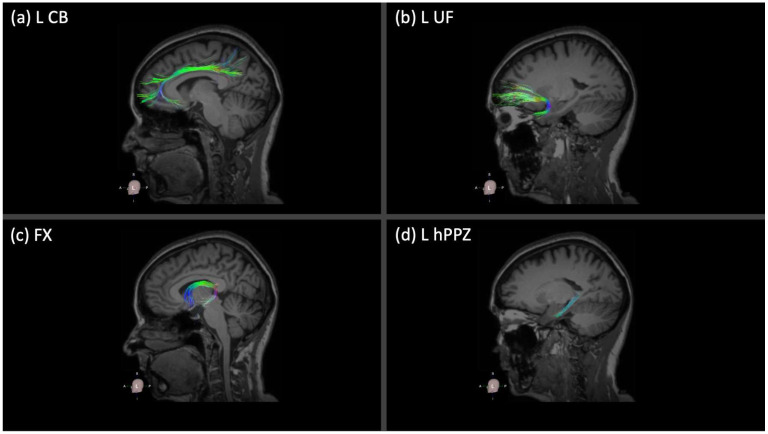
Three-dimensional representation of the reconstructed (**a**) L CB, (**b**) L UF, (**c**) Fx, and (**d**) L hPPZ, which are color-coded according to the distribution of the FA values along each tract and are projected over a T1 sequence. The reconstruction of the tracts has been performed in one of the participants of the study using the Brainance MD platform (Advantis Medical Imaging). L = left; CB = cingulum bundle; UF = uncinate fasciculus; Fx = Fornix; hPPZ = hippocampal perforant pathway zone; WM = white matter; FA = fractional anisotropy.

DTI data were processed using the Brainance MD DTI module (Advantis Medica Imaging, Eindhoven, The Netherlands), and tractography of major memory-related WM tracts was conducted using an advanced deterministic algorithm [51] and automated tractography pipeline based on an atlas-based approach for the selection of tracts’ regions of interest. All DTI data sets underwent motion and eddy-current correction with the scanner-registration tool as well as the co-registration protocol included in the Brainance MD DTI module before core analyses. In line with the purpose of the study, we focused on limbic WM tracts, and we reconstructed the following tracts: right and left cingulum bundle (CB), right and left uncinate fasciculus (UF), fornix (FX), and right and left hippocampal perforant pathway zone (hPPZ). Mean fractional anisotropy was extracted for each tract and used as the quantitative DTI measure of each tract. Mean values for bilaterally reconstructed tracts (i.e., CB, UF, hPPZ) were averaged for each tract, considering that no neurobiological explanation is currently available that supports a specific effect of food groups on the left or right hemisphere. Figure 1 demonstrates the 3D representation of the reconstructed WM tracts for a healthy individual in our group (for simplicity, only the left hemispheric tracts are shown for CB, UF, and hPPZ).

### 2.4. Dietary Intake Assessment

Dietary intake was assessed by four 24-h recalls using a modified version of the multi-pass method [52], which accurately evaluates energy and macronutrients in both sexes [53,54]. A trained registered dietitian (A.D.) asked all participants to report in detail all foods and beverages consumed the day before the assessment during the period covered between waking up in the morning and going to bed at night. To determine the usual intake more accurately throughout the week, three recalls were carried out during the week and one during the weekend. The recall day was not disclosed to the participants beforehand; thus, they were unable to modify their food in anticipation of the interview. Total energy and macronutrient intake were calculated per 2-h intervals using the dietary analysis software Nutritionist ProTM (version 4.2, 2007, Axxya Systems, Washington WA, USA). Traditional Greek food and recipes were added to the food database of the software. Recall data were grouped into individual food groups. The following food groups were included in the current analysis due to their potential association with cognition and, particularly, memory function, and as core food groups of the diets under investigation: [1] non-refined cereals (1 serv. = 30 g whole-grain bread, ½ cup whole-grain cereals, ½ cup whole-grain pasta or rice, etc.), [2] fruits (1 serv. = 1 medium fruit or 1 cup, ½ cup 100% fruit juice), vegetables (1 serv. = 1 cup raw vegetables or ½ cup cooked vegetables), and legumes (1/2 cup boiled legumes), [3] fish (1 serv. = 60 g cooked fish), [4] full-fat dairy (1 serv. = 1 cup full-fat milk, 1 cup yoghurt, 30 g full-fat cheese), [5] red meat and cold cuts (1 serv. = 60 g cooked red meat or cold cuts), and [6] alcohol (1 serv. = 330 mL beer, 150 mL wine, 30 mL other alcohol beverages).

### 2.5. Statistical Analysis

Categorical variables are presented as absolute values whereas continuous variables are presented as mean ± standard deviation (SD). Univariate linear regression models adjusted for age, sex, and education were applied to evaluate the association between memory performance (dependent variable) and FA values of WM tracts (independent variables) and verify meaningful further analyses within the study group. Following previous studies [55], the association between individual food groups and (a) memory performance as well as (b) integrity of memory-related WM tracts was assessed using univariate linear regression models, adjusted for age, sex, education, and calorie intake. The composite z-score of memory domain or the FA value of each WM tract were entered as dependent variables, and the food groups were entered as independent variables in univariate models. In case of a significant association between individual food group and FA value, supplementary analysis was conducted including Dax and Drad as dependent variables, in line with previous studies [47]. Multicollinearity was evaluated by examining the correlation matrix and multicollinearity diagnostics (i.e., tolerance, variance inflation factor). A *p*-value < 0.05 was defined as a statistically significant difference. All analyses were performed using IBM SPSS v.29.

## 3. Results

### 3.1. Descriptive Values for the Main Variables of the Study

In total, 117 non-demented individuals at baseline ALBION’s evaluation were included in the analysis. The study group main characteristics are presented in Table 1.

### 3.2. Association between Memory Performance and Integrity of Limbic WM Tracts

**Figure 2:**
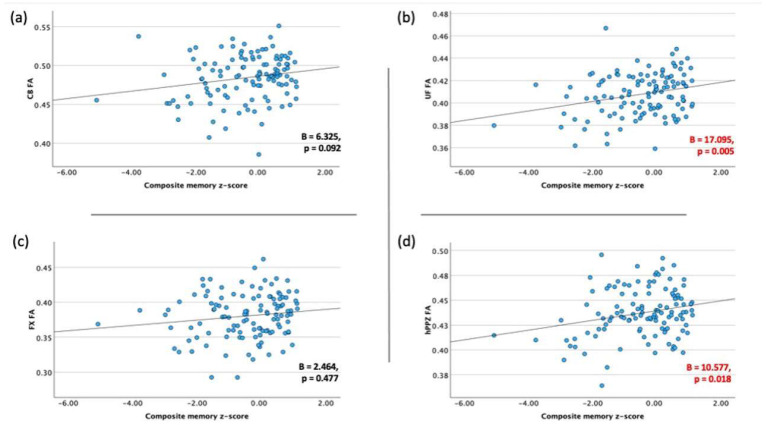
Scatter plots and lines of best fit for the association between composite memory z-score and FA values of limbic WM tracts, (**a**) CB, (**b**) UF, (**c**) FX and (**d**) hPPZ. CB = cingulum bundle; UF = uncinate fasciculus; FX = fornix; hPPZ = hippocampal perforant pathway zone; FA = fractional anisotropy; WM = white matter.

After adjusting for age, sex, and education, all correlations were within the expected direction, i.e., higher memory performance was associated with higher FA values of WM tracts (Figure 2). Significant associations were observed between composite memory z-score and UF FA (B = 17.095, *p* = 0.005) as well as hPPZ (B = 10.577, *p* = 0.018). The associations with CB (B = 6.325, *p* = 0.092) and FX (B = 2.464, *p* = 0.477) did not reach significance.

### 3.3. Food Groups and Integrity of Memory-Related (Limbic) WM Tracts

After adjusting for age, sex, education, and total energy intake, intake of full-fat dairy (B = −0.006, *p* = 0.029), as well as red meat and cold cuts (B = −0.007, *p* = 0.002), were negatively associated with PPZ FA (Table 2). Higher consumption of full-fat dairy as well as red meat and cold cuts were related to lower PPZ FA values. Further analysis on Dax and Drad did not reveal any significant associations. On the other hand, alcohol intake was positively associated with FX FA (B = 0.014, *p* = 0.009), with higher alcohol intake being related to higher FA values (Table 2). Supplementary analysis on Dax and Drad revealed that this association was mostly driven by Drad, since alcohol intake was significantly associated with FX Drad (B = −6.627 × 10^−5^, *p* = 0.047) rather than Dax (B = −7.721 × 10^−5^, *p* = 0.096). In a supplementary analysis, the association between red meat and cold cuts and hPPZ FA remained significant after false discovery rate (FDR) correction for multiple comparisons (p_FDR_ = 0.012), whereas the other two associations were marginally significant (alcohol and FX FA, p_FDR_ = 0.054; full-fat dairy and hPPZ FA, p_FDR_ = 0.087).

### 3.4. Food Groups and Performance on Memory-Related Neuropsychological Tests

After adjusting for age, sex, education, and calorie intake, none of the individual food groups was significantly associated with composite memory z-score (Table 3).

## 4. Discussion

Food intake can be considered as having a potential beneficial or harmful role on cognition and brain based on existing knowledge and examined in relation to disease risk [18]. Our study highlights that individual food groups may exert specific actions within WM tracts of the limbic system. By examining cognitive and neuroanatomical WM substrates of episodic memory, we identified that higher low-to-moderate alcohol intake was associated with higher FA of the FX whereas higher intake of full-fat dairy, as well as red meat and cold cuts, was associated with lower FA of the hPPZ. None of the food groups was associated with the performance on episodic memory tests.

### 4.1. Food Groups and Integrity of Limbic WM Tracts

**Figure 3:**
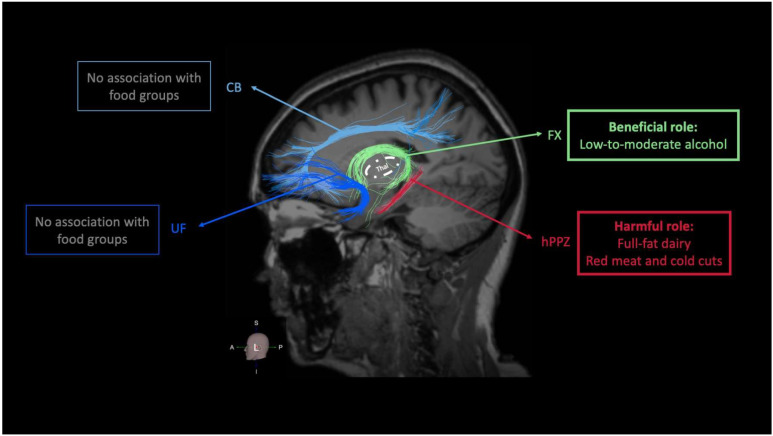
Summary of the associations between food groups and FA of limbic WM tracts. Different user-specific colors were selected for the reconstructed WM tracts for visualization purposes. FA = fractional anisotropy; WM = white matter; L = left; CB = cingulum bundle; UF = uncinate fasciculus; FX = fornix; hPPZ = hippocampal perforant pathway zone; Thal = thalamus.

Nutrition and dietary patterns may modulate structural brain changes, particularly in aging [56,57]. For example, a health-aware diet characterized by higher consumption of fruits and lower consumption of eggs, meat, and spirits, has been associated with higher global FA, indicating better global WM connectivity [58]. In addition, higher adherence to the Mediterranean diet (MeDi), a plant-based dietary pattern, has been associated with preserved WM microstructure [59]. Different food groups or individual components from various sources may exert region-specific actions within brain structures and WM connectivity. Yet, the field of dietary-related changes in WM connectivity is less well-characterized compared to changes in GM volume, cortical thickness, or WMH [56,57]. In our study, we found a positive association between intake of alcohol and FA values of the FX. Of note, alcohol consumption in our participants was defined as low-to-moderate and never exceeded the guidelines for normal alcohol use. On the other hand, we found an inverse correlation between FA of the hPPZ and intake of full-fat dairy as well as red meat and cold cuts. No associations were detected between food groups intake and integrity of CB or UF. From a neuroanatomical point of view, Pascalau and colleagues proposed that using the thalamus as a center of two rings, CB and UF belong to the outer ring of limbic WM tracts, whereas FX belongs to the inner ring [11]. Based on its anatomical location, the hPPZ can also be included in the inner ring. In our study, we found that the inner ring of the limbic WM tracts is mainly associated with the intake of specific food groups, i.e., alcohol, full-fat dairy, as well as red meat and cold cuts (Figure 3). Whether this pattern carries additional neurobiological meaning in terms of food-related associations needs to be addressed in future cross-sectional and longitudinal studies. A supplementary analysis on diffusivity values further indicated that the association between alcohol intake and FX FA was mostly driven by changes in FX Drad, since a positive association was found between alcohol consumption and FX Drad but not FX Dax. Several factors may explain changes in FA values in WM tracts [60]. Animal and human in vitro studies indicate that changes in Dax mainly reflect axonal injury whereas changes in Drad are representative of myelin damage [61]. The latter appears to increase Drad and reduce FA, and FA seems to primarily reflect Drad rather than Dax [62,63,64].

#### 4.1.1. Food Groups with a Potential Beneficial Role on Limbic WM Integrity: Non-Refined Cereals, Fruits, Vegetables, Legumes, and Fish

To the best of our knowledge, no study has examined the associations between non-refined cereals, fruits, vegetables, legumes, and fish and the integrity of limbic WM tracts. With regards to other structural measures related to memory-related volume and cortical thickness, in two recent cross-sectional studies, no significant association has been found between intake of non-refined cereals and hippocampal volume [34,44]. With regards to vegetables consumption, no significant association has been reported between vegetables intake and hippocampal volume [34,36,44]. Available studies on fruits and brain have also yielded mixed results for hippocampal volume, supporting either a negative association [37] or no association [34,36,44]. Fish consumption has been associated with higher hippocampal volume in one cross-sectional study [38], yet other cross-sectional [44] or longitudinal [36] studies did not find any association with hippocampal volume.

#### 4.1.2. Food Groups with a Potential Harmful Role on Limbic WM Integrity: Dairy, Red Meat, and Cold Cuts

No study so far has reported an association between dairy products, particularly full-fat dairy, and the integrity of limbic WM tracts or other memory-related GM or WM metric. On the other hand, there is no evidence regarding the role of red meat and products on the integrity of limbic WM tracts, and no other study is also available regarding general WM integrity and red meat. No associations were found between red meat consumption and hippocampal volume [34,36,44].

#### 4.1.3. Food Groups with a Dose- and Frequency-Specific Role on Limbic WM Integrity: Alcohol

In contrast to chronic heavy alcohol, studies on low-to-moderate alcohol intake and integrity of limbic WM tracts are scarce. In a previous study among Japanese non-alcohol-dependent participants, a significant association between lifetime alcohol consumption and increased mean diffusivity in the right amygdala was observed among female but not male subjects using two different approaches of statistical inferences (voxel-based and ROI-based analysis) [46]. In a longitudinal cohort study, the authors did not identify clusters with significant associations with alcohol consumption within the limbic WM network [43]. In another cross-section study where 12 major WM tracts were examined, an inverted U-shaped association was detected between alcohol consumption and FA in several tracts, including UF, inferior-fronto-occipital fasciculus, superior longitudinal fasciculus, the forceps minor, and the anterior thalamic radiation. FA increased with increasing alcohol consumption, with a peak at moderate alcohol intake, and then declined with heavier intake [47].

Inconsistent findings regarding the effect of low-to-moderate alcohol on macroscopic limbic-related GM and WM measurements are also present. With regards to hippocampal GM volume, moderate alcohol consumers during late life had larger hippocampal volume compared to late-life abstainers [41]. On the other hand, in a recent longitudinal study, no or light alcohol consumption was independently associated with larger hippocampal volume [44], highlighting the adverse brain outcomes of heavier alcohol consumption [43]. In another longitudinal study, higher alcohol consumption over a follow-up period of 30 years was associated with increased odds of hippocampal atrophy in a dose-dependent fashion; participants who consumed > 30 units of alcohol per week were at the highest risk compared to abstainers, and moderate drinkers had three times the odds of right hippocampal atrophy [43]. A protective role of light drinking over abstinence on hippocampal volume was not established [43].

#### 4.1.4. Potential Mechanisms Underlying the Association between Individual Food Groups and Integrity of Limbic WM Tracts

Our findings support a possible harmful role of full-fat dairy, red meat, and cold cuts on hippocampal PPZ integrity. Dairy products are rich in proteins, minerals, bioactive peptides, vitamin B12, and calcium, which may be beneficial for brain and cognition [65]. In addition, fermented dairy products may modulate gut microbiota and may also positively impact brain and cognition [66]. However, high-fat dairy products, high in saturated fatty acids, can have negative effects on the brain through several mechanisms, such as hyperinsulinemia, endothelial damage, oxidative stress, and inflammation [67,68,69]. The consumption of high-fat dairy products may also increase the amount of bile acids since a greater amount of bile is necessary to digest and absorb dietary fat, thus potentially influencing bile acid metabolism [70]. Of note, altered bile acids synthesis and metabolism have been reported in human [71] and animal [72] studies of Alzheimer’s disease, further supporting the role of gut-brain axis [73]. Red meat is a source of high-quality animal proteins, which are rather important for humans’ brain health, supporting the integrity of neuronal membranes and the brain cellular integrity and contributing to neurotransmitters biosynthesis (i.e., dopamine/epinephrine/norepinephrin and serotonin, respectively) based on the neutral amino acids tyrosine and tryptophan [74]. However, red meat, similarly to full-fat dairy products, contains saturated fatty acids, which are associated with higher risk of dementia and MCI, possibly due to oxidative stress and inflammation [75,76,77], as well as to elevated plasma total and low-density lipoprotein cholesterol concentrations, which are also associated with risk of Alzheimer’s disease [78], since cholesterol has a key role in β-amyloid production and deposition [79]. In our analysis, the meat group also includes meat products, such as cold cuts and sausages, that are high in nitrites, N-nitroso compounds, and sodium. The substances may exert an additional detrimental effect on brain health. Widespread exposure to nitrites and nitrosamines has been associated with DNA damage, oxidative stress, and cell death, and may have a negative effect on the pathogenesis of Alzheimer’s-type neurodegeneration [80]. Not only the presence of N-nitroso compounds in processed meats but also the presence of heterocyclic amines and polycyclic aromatic hydrocarbons during high-temperature cooking and grilling may negatively affect brain structure and function [81]. In addition, a high intake of saturated fatty acids has also been related to a lower level of brain-derived neurotrophic factor (BDNF), neuroplasticity, and cognitive performance [82]. BDNF plays an important role in maintaining synaptic plasticity in learning and memory, and depletion of BDNF has been linked with Aβ accumulation, tau phosphorylation, neuroinflammation, and neuronal apoptosis [83].

On the other hand, our findings support a possible protective role of low-to-moderate alcohol intake on the integrity of FX. Polyphenols are the major wine-related compounds, particularly of red wine [84]. Polyphenols exert neuroprotective and neurorescue effects not only through antioxidant activities but also via a combined ability to antagonize amyloid aggregation, suppress neuroinflammation, modulate signaling pathways, and decrease mitochondrial dysfunction [85]. For example, rerveratrol, a polyphenolic compound of red wine, has been found to show a potential dementia-prevention efficacy [86,87,88]. In addition, some beer compounds, such as hops (one of the four essential beer ingredients alongside barley, yeast, and water), may positively affect brain structure and function. Iso-α-acids (the main bitter components of beer) enhance hippocampus-dependent memory and prefrontal cortex-associated cognitive function via the activation of the vagus nerve and dopamine neurotransmission, while matured hop bitter acids (oxidized components with β-carbonyl moieties derived from aged hops) also enhance memory functions via the activation of the vagus nerve and norepinephrine or acetylcholine neurotransmission-mediated mechanisms. (For a detailed description of the effect of hop bitter acids on brain and cognition, see [89].) Furthermore, a protective association has also been reported between low-to-moderate consumption of alcohol, blood pressure, and cardiovascular diseases [90,91,92], as well as increased high-density lipoprotein (HDL) levels, which are associated with a reduced risk of atherosclerosis [93].

### 4.2. Food Groups and Episodic Memory

Episodic memory alters with age whereas amnestic deficits represent a core feature of typical AD, associated with medial temporal lobe pathology and impaired small- and large-scale brain networks [94,95]. In fact, higher adherence to specific dietary patterns, such as the MeDi, has been linked to better memory performance [96], reduced risk of developing MCI and reduced risk of MCI conversion to AD [97], as well as reduced risk of developing AD [98]. Of note, the beneficial effect of MeDi in episodic memory and specific memory processes, such as immediate or delayed recall, has been identified mostly in longitudinal observational cohort studies and to a lesser extent in randomized controlled trials [18,99]. However, the association between the intake of specific food groups and episodic memory function is less well-characterized since previous studies have identified mixed and varied by study design evidence [18,100], and the main findings often include screening measures (e.g., MMSE) or general cognitive functioning instead of specific episodic memory scores. In our sample of 117 community-dwelling individuals, we did not identify any significant associations between memory performance and the consumption of (1) non-refined cereals, (2) fruits, vegetables, and legumes, (3) fish, (4) full-fat dairy, (5) red meat and cold cuts, and (6) alcohol. Methodological differences with previous studies that found significant associations described below may account for the current findings.

#### 4.2.1. Food Groups with a Potential Beneficial Role on Episodic Memory: Non-Refined Cereals, Fruits, Vegetables, Legumes, and Fish

To the best of our knowledge, there are no studies on the effects of non-refined cereals’ consumption on adult episodic memory function. In broader terms of cognitive status, consumption of whole-grain cereal has been associated with higher scores in the MMSE screening tool in women older than 60 years [101], whereas in the Hellenic Longitudinal Investigation of Aging and Diet (HELIAD) Study, a positive association was found between intake of non-refined cereals and the composite cognitive z-score [96].

The consumption of fruits and vegetables, fruits alone, as well as fruits with high vitamin C and vegetables, has been positively associated with adults’ verbal memory in the 13-year Supplementation en Vitamines et Mineraux Antioxydants 2 (SU.VI.MAX 2) prospective study [19]. In a previous study with community-dwelling elderly in China, green vegetables intake was positively associated with higher verbal episodic memory scores and reduced the risk for cognitive impairment by almost 20% [20]. However, based on the most recent meta-analysis, the intake of fruits and vegetables was significantly associated with the prevalence of cognitive impairment and dementia in general, but there was no association with the prevalence of AD particularly [102]. On the other hand, high tofu consumption, a soy-based food, has been associated with impaired memory and verbal learning in old age [20,103,104].

The long-term beneficial effects of fish consumption on cognition in older adults have been shown in several studies [18,100]. The consumption of ≥ four portions per week versus < one portion per week of fish was associated with a lower rate of memory decline in five cohorts of older participants, whereas participants with a consumption of at least four portions of fish per week have memory scores comparable to those who were 4 years younger [21]. Of note, no association between fish consumption and cognitive changes have been reported in adults aged 55–64 years [105].

#### 4.2.2. Food Groups with a Potential Harmful Role on Episodic Memory: Dairy, Red Meat, and Cold Cuts

Few studies have examined the association between dairy products and cognitive dysfunction or dementia, whereas specific results on full-fat dairy products are scarce [18,100]. In the longitudinal ARIC study, the intake of more than one glass of milk/day between the ages of 45 years and 64 years was associated with a 10% decline in global cognitive status and memory 20 years later [106]. Similarly, by analyzing data from the SU.VI.MAX cohorts of older adults, Park and Fulgoni found that total dairy product intake was not associated with cognitive function, but milk intake was negatively associated with verbal memory performance [22]. In a previous study including older Australian men, the regular use of full-cream milk was inversely associated with successful mental health aging [107]. However, in the Maine-Syracuse Longitudinal Study, individuals with the highest consumption of dairy products had the best performance on global cognition, visuospatial memory, and executive functions compared with individuals with rare consumption of dairy foods [23]. Furthermore, in another study, the incidence of AD was reduced in participants aged 60 years or older with high consumption of milk and dairy products [24].

There is mixed evidence on the association between red meat consumption and cognition in general or episodic memory in particular [108,109]. In some longitudinal cohort studies, no significant association has been found between red and processed meat consumption and mean score of global cognition or verbal memory or incidence of AD [25,26,27]. However, in the largest prospective study that examines the association between meat and cognitive function in approximately 500,000 participants, each additional portion per week of processed red meat (one more slice of ham, two more slices of bacon, or one more sausage) was associated with reduced cognitive function, including fluid intelligence and memory performance, and would increase the risk of dementia by more than 40% over an average 8 years of follow-up [30]. Of note, the consumption of unprocessed poultry was not associated with dementia risk, whereas the consumption of unprocessed red meat had modest protective association with cognitive outcomes [30]. An inverse correlation between processed meat (including but not limited to processed red meat) and cognitive performance (including but not limited to episodic memory scores) has also been reported in another longitudinal study [28]. In the Newcastle (UK) 85+ Cohort Study, there was an association between a dietary pattern high in red meat and poor cognitive performance in a large sample of 791 adults over a 5-year follow-up [110]. On the other hand, in the Shanghai Women’s Health Study and Shanghai Men’s Health Study (SWHS and SMHS), high red meat intake was associated with a lower likelihood of memory impairment [29]. Furthermore, in the Uppsala Seniors cohort study, a low consumption of meat and meat products was associated with better cognition in 194 cognitively healthy participants [39]. Additionally, in the Maine-Syracuse Longitudinal Study, higher intake of meat was prospectively associated with higher cognitive scores in 333 participants free of dementia and stroke [111].

#### 4.2.3. Food Groups with a Dose- and Frequency-Specific Role on Episodic Memory: Alcohol

With regards to alcohol, it is well-established that chronic, heavy alcohol consumption causes alcohol-related brain damage [112] and cognitive deficits [113,114]. Observation studies often report a “U”-shaped relationship between alcohol consumption and cognitive outcomes, including specific and all-cause dementias; low-to-moderate alcohol consumption is often associated with lower risk of dementia compared to abstention and heavy drinking [115]. In the Nurses’ Health Study of approximately 11,000 participants who were followed up for 2 years, moderate drinkers showed better general cognitive function and higher performance on verbal memory tests and had slower rates of cognitive decline compared with non-drinkers [31]. In animal studies, chronic low-dose ethanol consumption improves learning and memory in rats, and this association is mediated by sufficient levels of NMDA receptors’ expression in the hippocampus [116]. Other studies found no association between low-to-moderate alcohol consumption and cognitive outcomes [117].

### 4.3. Research and Clinical Considerations

Higher alcohol intake was associated with higher FA of the FX. Higher FA in the FX could suggest more coherent or denser WM pathways, potentially indicating a preservation or even enhancement of structural integrity in individuals with higher alcohol consumption. However, the functional implications of this finding, given the typically negative effects of alcohol on brain health, warrant further investigation. Higher intake of full-fat dairy, red meat, and cold cuts was associated with lower FA of the hPPZ. Lower FA in the hPPZ suggests a reduction in the integrity or coherence of these WM tracts. This could imply potential negative impacts on the structural connectivity within the hippocampus, a key region involved in episodic memory processes. These dietary components might contribute to detrimental changes in brain structure related to episodic memory. On the other hand, no significant association was found between individual food groups intake and FA of the CB or UF. We could hypothesize that the CB and the UF may be less susceptible to dietary influences, or the effects might be more subtle and not detected in this study. Despite significant associations between individual food groups and WM integrity, these did not translate to significant associations between food groups intake and episodic memory performance. This could indicate that the structural changes observed might not be sufficient to impact cognitive function, or that other compensatory mechanisms have already emerged to compensate for structural changes. Structural alterations in WM might reflect an intermediate phenotype before cognitive changes are evident. Alterations in WM integrity may contribute to cognitive changes due to progressive “disconnection” between cortical areas [118], and these alterations may serve as a potential beneficial feature for capturing additional or complementary markers of early degeneration before the onset of cognitive alterations. Patients with MCI may experience decreased WM integrity compared to normal individuals, abnormalities that have been related to cognitive decline [119]. Studies in MCI have shown that microstructural WM changes identified through DTI may not be apparent using standard anatomical imaging [120,121,122], and that changes in DTI metrics of specific limbic WM tracts may serve as a reliable marker of the onset of MCI [123]. Changes in WM integrity have also been proven as the most sensitive individual marker to discriminate MCI from healthy controls and classify MCI from AD when GM density, FA values, and metabolites’ ratios were simultaneously examined [124]. Hippocampal atrophy precedes symptoms in individuals with AD by several years [125], and a similar phenomenon has been highlighted in alcohol-related brain and cognitive changes [43]. Of note, similar findings of altered WM integrity before the onset of cognitive changes have been reported in other CNS pathologies, including multiple sclerosis [126] and Parkinson’s disease [127]. Not only WM changes detected through DTI may precede and predict volume loss [128], but also it appears that carriers of some Alzheimer’s disease risk genes show differences on WM microstructure as young adults, decades before the typical age of onset of dementia [129]. Therefore, WM microstructural changes may serve as a potential beneficial feature for capturing additional or complementary markers of early degeneration. Considering that nutritional factors play a pivotal role in brain aging [18] and that few studies are done on specific food groups and cognitive and brain health [100], our findings add to the growing body of evidence supporting the role of specific food groups on integrity of specific limbic WM tracts. Our findings also fit with a universal, healthy reference diet proposed by the EAT–Lancet Commission that consists of whole grains, fruits, vegetables, nuts, legumes, unsaturated oils, low to moderate amounts of seafood and poultry, and no or low red meat, processed meat, added sugar, refined grains, and starchy vegetables, and that could promote the absence of disease and optimize physical, mental, and social well-being [100].

### 4.4. Strengths, Limitations, and Future Directions

Memory function was assessed by using standardized neuropsychological tests as part of a comprehensive assessment whereas the analysis of DTI data and WM tractography were conducted using an atlas-based automated approach, minimizing rater-related errors of manual tractography. Middle-aged and older adults often have established dietary patterns [130], and non-demented older adults can provide valid dietary-related self-reports [131] that often remain relatively stable over time [132]. In addition, widely used and accepted ways of assessing dietary habits, such as 24-h recall and Food Frequency Questionnaires, provide dietary data that could reflect long-term exposure variables [133,134]. Therefore, it is not inconceivable that dietary habits reported in our study could represent long-term nutritional exposures. However, a more reliable conclusion regarding a potentially prolonged nutritional exposure would require a longitudinal design over a long period with multiple dietary assessments. Although demographic variables and calorie intake were assessed as covariates, we cannot exclude the possibility that other important confounders were omitted. We herein examined the association between food intake and “dry biomarkers” (i.e., neuroimaging and cognitive metrics). These findings may be altered considering the additive role of “wet biomarkers”, such as plasma [135] and cerebrospinal fluid biomarkers [136,137,138], which are known to contribute to AD diagnosis and the study of the normal aging-dementia continuum. A future comprehensive review paper may further evaluate the association between individual food groups and neuroimaging biomarkers. In addition, we did not examine stratified-related models, e.g., age-stratification, sex-stratification, education-stratification, etc., considering the sample size of the group. Furthermore, in the absence of specific hypotheses on hemispheric asymmetries related to food groups consumption, we did not separately examine the association between food groups and left or right WM tracts, but we looked at the average of left and right DTI metrics. Future studies in larger sample sizes can focus on each hemisphere to further address this issue. Due to the sample size of our group, we did not evaluate further models between low and high consumers of different food groups. The association between temporal dimensions of these food groups and anatomical substrate of memory function may be clinically significant and may need further investigation in future studies with larger sample sizes, since it has been found that temporal eating patterns are associated with cognitive performance and, in particular, memory performance [139]. Moreover, our analyses were based on the baseline data of the observational longitudinal ALBION study [49,50]. Thus, our findings can currently provide associations but not causality, which can be further examined in our prospectively acquired data as well as in other longitudinal studies. Considering that brain changes may be already present in midlife before evidence of severe cognitive decline at old age [140], and that nutrition is a modifiable factor exposure [141], further research is therefore required to better elucidate the complex association between individual food groups and WM integrity both in cross-sectional and longitudinal designs, including additional MRI modalities, such as functional MRI, to test both structural and functional connectivity within the limbic network.

## 5. Conclusions

The study documented an association between individual food groups consumption and limbic WM tracts. Higher intake of alcohol was associated with better integrity of the FX, whereas higher intake of full-fat dairy as well as red meat and cold cuts was associated with worse integrity of the hPPZ. None of the food groups was associated with memory performance. Considering that nutritional factors are modifiable across the life span, our findings may have significant implications in aging and nutritional neuroscience, highlighting the association between food groups and brain biomarkers and emphasizing the potential need for dietary recommendations to support brain health in clinical settings.

## Figures and Tables

**Table 1:** Descriptive values for the main demographic, cognitive, neuroimaging, and dietary data of the study group.

Study Variables	Descriptive Measures
Demographic data	
Age (years)	63.58 ± 9.19
Sex (M/F)	39/78
Education (years)	13.39 ± 3.78
Cognitive data	
MMSE	28.22 ± 1.98
ACE-R total	90.90 ± 7.15
Memory composite z-score	−0.47 ± 1.17
Neuroimaging data (DTI)	
CB FA	0.48 ± 0.03
CB Dax (×10^−3^)	1.27 ± 0.04
CB Drad (×10^−3^)	0.56 ± 0.04
UF FA	0.41 ± 0.02
UF Dax (×10^−3^)	1.26 ± 0.06
UF Drad (×10^−3^)	0.65 ± 0.05
FX FA	0.38 ± 0.03
FX Dax (×10^−3^)	1.97 ± 0.26
FX Drad (×10^−3^)	1.13 ± 0.19
hPPZ FA	0.44 ± 0.02
hPPZ Dax (×10^−3^)	1.26 ± 0.05
hPPZ Drad (×10^−3^)	0.62 ± 0.04
Dietary data	
Total energy intake (kcal/day)	1723.64 ± 492.20
Non-refined cereals (servings/day)	1.70 ± 1.66
Fruits, vegetables, legumes (servings/day)	4.22 ± 2.66
Fish (servings/day)	0.43 ± 0.62
Full-fat dairy (servings/day)	0.73 ± 0.87
Red meat and cold cuts (servings/day)	0.99 ± 1.09
Alcohol (servings/day)	0.26 ± 0.55

Notes. Descriptive values on sex are presented as absolute values whereas descriptive values on other variables are presented as mean ± standard deviation. M/F = male/female; MMSE = Mini-Mental State Examination; ACE-R = Addenbrooke’s Cognitive Examination—revised; DTI = diffusion tensor imaging; FA = fractional anisotropy; CB = cingulum bundle; UF = uncinate fasciculus; FX = fornix; hPPZ = hippocampal perforant pathway zone; kcal = kilocalories.

**Table 2:** Univariate linear regression models of food groups and FA value of memory-related (limbic) WM tracts, adjusted for demographic data and total energy intake.

Independent Variable: Food Groups	Dependent Variable: FA Value of WM Tract			
	CB FA	UF FA	FX FA	hPPZ FA
Non-refined cereals	B = 3.078E-5 [CI: −0.003 to 0.003], *p* = 0.985	B = 0.002 [CI: 0.000 to 0.004], *p* = 0.114	B = 0.001 [CI: −0.002 to 0.005], *p* = 0.551	B = 0.002 [CI: −0.001 to 0.005], *p* = 0.164
Fruits, vegetables, legumes	B = 0.000 [CI: −0.002 to 0.002], *p* = 0.864	B = −0.001 [CI: −0.002 to 0.001], *p* = 0.351	B = −0.001 [CI: −0.003 to 0.002], *p* = 0.597	B = 0.000 [CI: −0.002 to 0.002], *p* = 0.797
Fish	B = 0.004 [CI: −0.004 to 0.012], *p* = 0.359	B = 0.002 [CI: −0.003 to 0.007], *p* = 0.421	B = 0.000 [CI: −0.009 to 0.009], *p* = 0.965	B = 0.004 [CI: −0.003 to 0.011], *p* = 0.315
Full-fat dairy	B = −0.002 [CI: −0.009 to 0.004], *p* = 0.515	B = −0.003 [CI: −0.007 to 0.001], *p* = 0.125	B = −0.001 [CI: −0.008 to 0.006], *p* = 0.732	B = −0.006 [CI: −0.011 to −0.001], *p* = **0.029**
Red meat and cold cuts	B = −0.003 [CI: −0.008 to 0.002], *p* = 0.276	B = −0.002 [CI: −0.005 to 0.001], *p* = 0.229	B = −0.002 [CI: −0.008 to 0.004], *p* = 0.473	B = −0.007 [CI: −0.011 to −0.003], *p* = **0.002**
Alcohol	B = 0.000 [CI: −0.010 to 0.010], *p* = 0.968	B = 0.003 [CI: −0.003 to 0.009], *p* = 0.337	B = 0.014 [CI: 0.004 to 0.025], *p* = **0.009**	B = 0.006 [CI: −0.002 to 0.014], *p* = 0.141

Notes. WM = white matter; FA = fractional anisotropy; CB = cingulum bundle; UF = uncinate fasciculus; FX = fornix; hPPZ = hippocampal perforant pathway zone; CI = confidence interval at 95%. Bold *p*-values values indicate a significant association between the individual food group and FA value of the WM tract at *p* < 0.05.

**Table 3:** Univariate linear regression models of food groups and memory function, adjusted for demographic data and calorie intake.

Independent Variable: Food Groups	Dependent Variable: Composite Memory z-Score
Non-refined cereals	B = 0.002 [CI: −0.127 to 0.131], *p* = 0.974
Fruits, vegetables, legumes	B = −0.010 [CI: −0.092 to 0.073], *p* = 0.821
Fish	B = 0.192 [CI: −0.140 to 0.524], *p* = 0.253
Full-fat dairy	B = −0.129 [CI: −0.384 to 0.125], *p* = 0.317
Red meat and cold cuts	B = 0.129 [CI: −0.083 to 0.342], *p* = 0.231
Alcohol	B = 0.047 [CI: −0.350 to 0.445], *p* = 0.814

Notes. CI = confidence interval at 95%.

## Data Availability

Dataset available on reasonable request from the authors.
